# How social factors relate to arthritis risk in Chinese older adults: population-based evidence

**DOI:** 10.3389/fpubh.2025.1604582

**Published:** 2025-06-18

**Authors:** Liang Xu, Jiangning Fu, Boyu Zhai, Jing Li

**Affiliations:** ^1^Department of Orthopedic, The Second Affiliated Hospital of Harbin Medical University, Harbin, China; ^2^School of Nursing, University of British Columbia, Vancouver, BC, Canada; ^3^Center on Aging Psychology, CAS Key Laboratory of Mental Health, Institute of Psychology, Chinese Academy of Sciences, Beijing, China; ^4^Department of Psychology, University of Chinese Academy of Sciences, Beijing, China; ^5^Department of Geriatrics, The Second Affiliated Hospital of Harbin Medical University, Harbin, China

**Keywords:** social factors, older adults, arthritis, social participation, emotional support

## Abstract

**Background:**

Arthritis prevalence is rising among older adults globally, including in China, where the aging population is increasing. Social factors, such as social networks, participation, and support, have been associated with inflammation response. However, limited research has explored how these factors affect arthritis in Chinese older adults. This study aimed to examine the association between social factors and arthritis risk using data from the 2017–2018 Chinese Longitudinal Healthy Longevity Survey (CLHLS).

**Methods:**

A total of 15,854 individuals aged 60 and above were included. Arthritis status was assessed through self-reports, and key social factors—social network size, social participation, and social support were measured. Binary logistic regression models were used to analyze associations, adjusting for demographic and health-related variables.

**Results:**

A larger social network was associated with a lower risk of arthritis (OR = 0.963, *p* = 0.015). Higher social participation, however, was associated with a higher risk (OR = 1.027, *p* = 0.007). Emotional support demonstrated a protective effect (OR = 0.963, *p* = 0.006), while instrumental support showed no significant impact. A significant interaction between social participation and emotional support (*p* = 0.008) indicated that emotional support mitigates the elevated risk of arthritis associated with high social participation.

**Conclusion:**

Social factors play a crucial role in arthritis risk among older adults. While broader social networks and emotional support appear beneficial, increased social participation may contribute to higher risk. These findings highlight the need for tailored interventions to promote healthy aging.

## 1 Introduction

The prevalence of arthritis has shown a significant increasing trend among adults aged 60 and older worldwide (1, 2). China, with a high proportion of older adults, faces a growing burden of arthritis, with the number of affected individuals rising to 61.2 million (3). This trend has substantially increased healthcare costs and diminished the quality of life among the aging population.

Although arthritis has a multifactorial pathogenesis, chronic inflammation is a key contributing factor (4, 5). Existing research has demonstrated a close association between social factors and inflammation risk. A 10-year longitudinal study conducted in the U.S. found that social support can reduce the risk of inflammation, whereas social stress increases it (6). Additionally, high-quality social relationships have been shown to mitigate inflammation risk (7). Social support, social interactions, and the size of social networks play a crucial moderating role in the relationship between inflammation and depressive symptoms (8). Moreover, among individuals with arthritis, lower levels of social support are significantly associated with a higher likelihood of disability (9).

Accordingly, this study aims to investigate the association between social factors and arthritis among older adults using large-scale nationwide data from China. These findings may inform self-care strategies for older adults and guide policymakers in optimizing social engagement interventions.

## 2 Materials and methods

### 2.1 Data and study participants

This study utilized cross-sectional data from the 2017–2018 Chinese Longitudinal Healthy Longevity Survey (CLHLS), a nationally representative survey conducted in half of the randomly selected counties/cities across 23 of China’ s 31 provinces. The CLHLS data are publicly available for research purposes upon application approval. The survey collected comprehensive data on physical and mental health, living conditions, socio-economic characteristics, and lifestyle through structured questionnaires administered by trained interviewers during home visits. The study protocol was approved by the Ethics Committee of Peking University Health Science Center (Ethical approval number: IRB00001052-13074), and written informed consent was obtained from all participants or their legal representatives.

Of the 15,874 participants, 20 individuals under 60 years of age were excluded. To address missing data while preserving sample size and minimizing bias, multiple imputation was conducted using SPSS (version 29.0), based on the fully conditional specification (FCS) method. Variables with 5–20% missing data, including several demographic, physical health, and psychosocial measures, were imputed. Variables with less than 5% missingness were not imputed. All imputation models included theoretically relevant sociodemographic and health-related predictors. Five imputed datasets were generated and pooled for analysis. After imputation, missing rates for all variables were reduced to below 5%. The final sample for analysis included 15,854 participants.

### 2.2 Assessment of social factors

In this study, social factors were assessed across three dimensions: social network size, social participation, and social support.

Social network size was measured by the number of people living with the participant, excluding the participant themselves. A larger number indicates a larger social network.

Social participation was assessed through participants’ engagement in daily social activities, including four items: participation in Tai Chi, participation in square dancing, visiting friends’ homes, and engaging in organized social activities. Each item was rated on a scale from 1 to 5, where 1 represents “never,” 2 “occasionally,” 3 “monthly,” 4 “weekly,” and 5 “almost daily.” The total social participation score was the sum of these four items, with a higher score indicating greater social participation.

Social support was divided into emotional support and instrumental support. Emotional support was assessed with the question, “If you have concerns or thoughts, who would you talk to first?” Instrumental support was assessed with the question, “If you encounter problems or difficulties, who would you seek help from first?” Participants could nominate up to two individuals and specify their relationship with each. Responses were re-coded according to the concentric circle model of social networks (10), where 1 indicates no support, 2 indicates friends, 3 indicates extended family (grandchildren, nephews, other relatives), and 4 indicates core family members (spouse and children) (11, 12). The total scores for emotional and instrumental support were calculated by weighting the nominated relationships, with higher scores indicating stronger social support.

### 2.3 Assessment of arthritis

In this survey, the presence of arthritis was determined based on a self-reported question: “Do you currently have arthritis?” For this study, the responses were recoded as 1 = Yes and 0 = No.

### 2.4 Covariates

The covariates in this study included three parts: demographic characteristics, physical health, and psychological indicators. Demographic characteristics included age, gender, years of education, and annual household income. Physical health included BMI, waist-to-hip ratio, disability in ADL (whether health issues had restricted daily activities in the past 6 months), and subjective health (how the participant perceived their current health status). Psychological indicators included the Generalized Anxiety Disorder-7 (GAD-7) (13), the Center for Epidemiologic Studies Depression Scale-10 (CESD-10) (14), and the Community Screening Instrument for Dementia (CSI-D) (15).

### 2.5 Statistical analysis

Continuous variables were presented as means (standard deviations), and categorical variables were presented as numbers and percentages. Chi-square tests or t-tests were employed to compare differences between participants with and without arthritis. All statistical tests were two-tailed, and *p* < 0.05 was considered statistically significant. A binary logistic regression model was used to analyze the relationship between social factors and arthritis status. All statistical analyses were performed using SPSS version 29.0 software.

## 3 Results

### 3.1 Basic information

A total of 15,854 older adults aged 60 and above were included in the study, with 1727 participants reporting arthritis and 14,127 without arthritis. Significant differences were observed between the two groups across demographic, physical health, psychological, and social indicators (Table 1).

**Table 1:** Demographic and general characteristics of participants by arthritis status.

Variable	Total Mean ± SD or *N* (%)	With arthritis Mean ± SD or *N* (%)	Without arthritis Mean ± SD or *N* (%)	*t/χ^2^*	*p*-value
*N*	15,854	14,127	1727		
Age (years)	85.48 ± 11.68	83.71 ± 11.15	85.70 ± 11.72	−6.933	<0.001
Gender (Female)	8,942 (56.40)	1,104 (63.93)	7,838 (55.48)	44.615	<0.001
Years of education	3.31 ± 4.15	4.07 ± 4.57	3.21 ± 4.09	7.447	<0.001
Annual household income (Yuan, >30,000)	8,298 (52.34)	958 (55.47)	7,340 (51.96)	7.621	0.006
BMI (kg/m^2^)^a^	22.38 ± 3.82	23.06 ± 4.06	22.30 ± 3.79	7.325	<0.001
Waist-to-hip ratio	0.92 ± 0.08	0.92 ± 0.08	0.92 ± 0.08	0.954	0.340
Disability in ADL (Yes)^b^	5,878 (37.08)	741 (42.91)	5,137 (36.36)	28.246	<0.001
Subjective health	3.43 ± 0.90	3.16 ± 0.90	3.46 ± 0.89	−13.074	<0.001
Anxiety score	1.37 ± 2.77	1.86 ± 3.09	1.31 ± 2.72	6.890	<0.001
Depression score	20.81 ± 5.77	21.60 ± 5.95	20.71 ± 5.74	5.796	<0.001
CSI-D score^c^	4.81 ± 2.82	5.03 ± 2.75	4.79 ± 2.82	3.460	<0.001
Number of cohabitants	2.43 ± 1.91	2.30 ± 1.83	2.44 ± 1.92	−2.958	0.003
Social participation	2.66 ± 2.94	2.93 ± 3.11	2.63 ± 2.92	3.817	<0.001
Emotional support	6.06 ± 2.09	5.93 ± 2.13	6.07 ± 2.09	−2.542	0.011
Instrumental support	7.10 ± 1.43	7.11 ± 1.45	7.10 ± 1.43	0.365	0.715

a BMI, Body Mass Index.

b ADL, Activities of Daily Living; cognition.

c CSI-D, Community Screening Instrument for Dementia.

Demographic Characteristics: Participants with arthritis were younger, had a higher proportion of females, more years of education, and higher annual household income compared to those without arthritis.

Physical Health: The arthritis group had a higher BMI, worse ADL function, and lower subjective health scores. However, no significant difference was found in waist-to-hip ratio.

Psychological Indicators: Participants with arthritis exhibited higher levels of anxiety and depression but better cognitive function.

Social Indicators: The arthritis group had fewer cohabitants, greater social participation, and slightly lower emotional support, though no significant difference was found in instrumental support (Table 2).

**Table 2:** Variable assignment and measured ranges of social factors and other influencing factors in arthritis among older adults.

Variable	Type	Assignment/range
Age	Continuous	Range: 60–117 years
Gender	Categorical	0 = Female; 1 = Male
Years of education	Continuous	Range: 0–22 years
Annual household income	Categorical	0 = ≤30,000 Yuan; 1 = > 30000Yuan
BMI	Continuous	Range: 15.01–39.82 kg/m^2^
Waist-to-hip ratio	Continuous	Range: 0.44–1.38
Disability in ADL	Categorical	0 = No; 1 = Yes
Subjective health	Ordinal	1 = Very poor; 2 = Poor; 3 = Fair; 4 = Good; 5 = Very good.
Anxiety score	Continuous	Range: 0–21
Depression score	Continuous	Range: 9–43
CSI-D score	Continuous	Range: 0–7
Number of cohabitants	Continuous	Range: 0–21
Social participation	Continuous	Range: 0–20
Emotional support	Continuous	Range: 1–8
Instrumental support	Continuous	Range: 2–8

### 3.2 Association between social factors and arthritis

A binary logistic regression analysis was performed to explore the relationship between social factors and arthritis in older adults, adjusting for demographic and health-related covariates (Supplementary Table 1). The results, shown in Table 3, revealed that social network size (number of cohabitants) was inversely associated with arthritis risk, while social participation was positively associated. Emotional support had a protective effect, but instrumental support did not show a significant association. The goodness-of-fit of the model was confirmed by the Hosmer-Lemeshow test (*χ^2^* = 7.706, *df* = 8, *p* = 0.463).

**Table 3:** Binary logistic regression analysis of social factors associated with arthritis in older adults.

Variable	B	SE	Wald	*p*-value	OR [Exp(B)]	95% CI for OR
Number of Cohabitants	−0.038	0.016	5.879	0.015	0.963	0.934–0.993
Social Participation	0.026	0.010	7.210	0.007	1.027	1.007–1.047
Emotional Support	−0.038	0.014	7.642	0.006	0.963	0.937–0.989
Instrumental Support	0.014	0.021	0.486	0.486	1.014	0.974–1.056

OR, Odds Ratio; CI, Confidence Interval. The full model, including all covariates, is presented in Supplementary Table 1.

Additional exploratory analyses were conducted to distinguish between sedentary (e.g., chatting, playing cards) and non-sedentary (e.g., Tai Chi, square dancing) social activities. However, neither category showed a significant association with arthritis risk after adjusting for covariates (*p* = 0.082 and *p* = 0.713), suggesting that the overall association was not primarily driven by activity type. Therefore, we retained the composite social participation score in our main analysis.

### 3.3 Interaction effects of social factors on arthritis risk

Building upon the analysis presented in section 3.2, we further explored the interaction between social factors and the risk of arthritis. Binary logistic regression models were employed to examine the interactions between social participation, number of cohabitants, and emotional support (see Table 4).

**Table 4:** Interaction effects of social factors on arthritis in older adults.

Variable	B	SE	Wald	*p*-value	OR [Exp(B)]	95% CI for OR
Social Participation × Number of Cohabitants	−0.005	0.005	1.105	0.293	0.995	0.985–1.005
Social Participation × Emotional Support	−0.010	0.004	7.030	0.008	0.990	0.982–0.997
Number of Cohabitants × Emotional Support	−0.006	0.007	0.826	0.363	0.994	0.981–1.007

OR, Odds Ratio; CI, Confidence Interval.

As shown in Table 4, the interaction between social participation and emotional support was significantly associated with arthritis risk. Specifically, the odds ratio (OR) for this interaction was 0.990 (*p* = 0.008), indicating that the combined effect of social participation and emotional support may influence the likelihood of arthritis. However, no significant interactions were found between social participation and number of cohabitants, or between number of cohabitants and emotional support, with *p*-values of 0.293 and 0.363, respectively.

**Figure 1:**
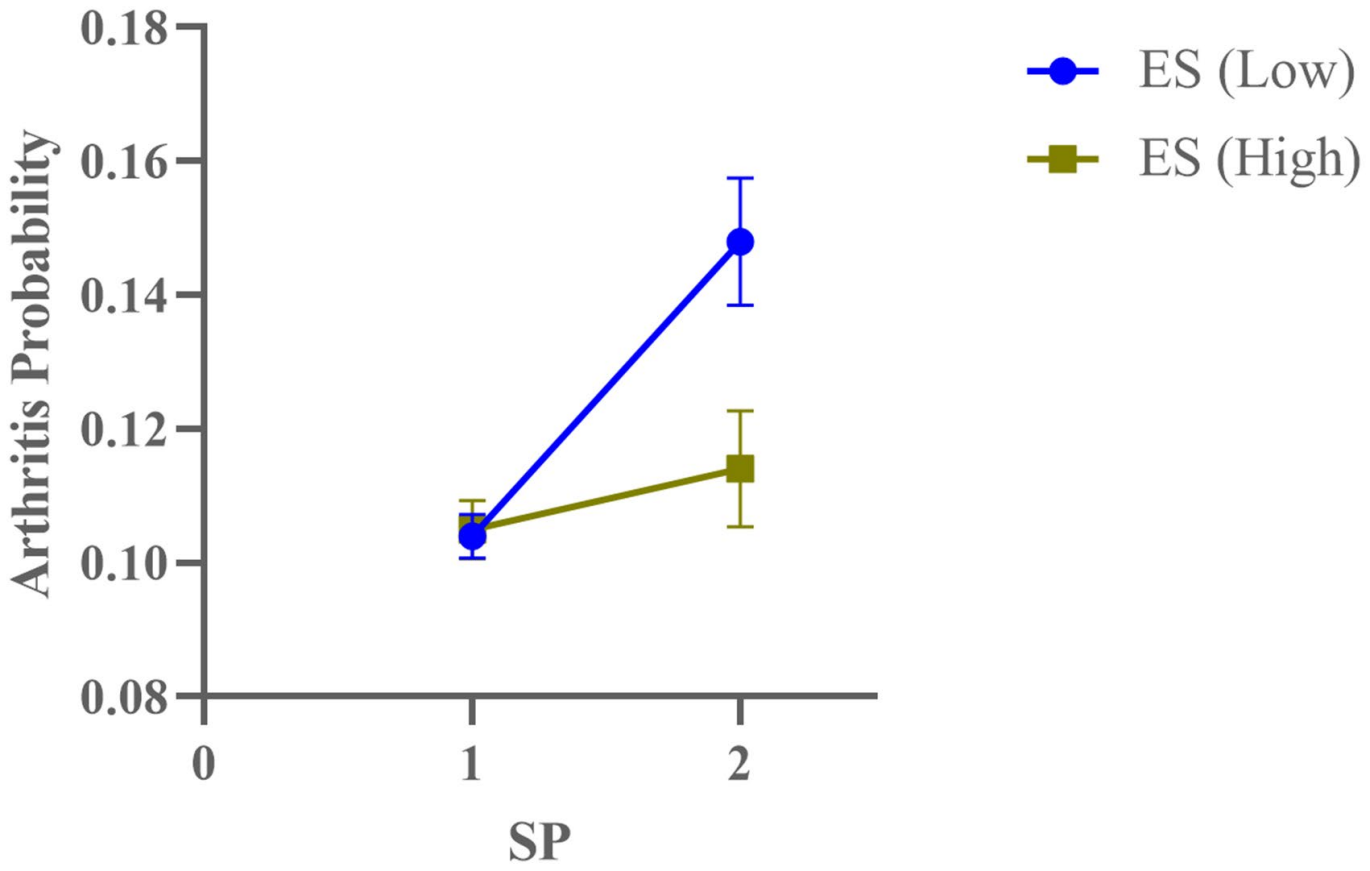
Interaction between social participation and emotional support on arthritis risk in older adults. Error bars represent standard errors. ES, Emotional Support; SP, Social Participation.

To further illustrate these interaction effects, Figure 1 displays the predicted probabilities of arthritis risk under different combinations of social participation and emotional support. In Figure 1, social participation and emotional support were dichotomized into high and low groups based on their mean values. The figure shows that individuals with low levels of both social participation and emotional support had the lowest probability of arthritis risk (0.104). In contrast, individuals with high social participation but low emotional support exhibited a higher risk of arthritis (0.148). Interestingly, increasing emotional support had a minimal effect on arthritis risk for individuals with low social participation (from 0.104 to 0.105), but a more pronounced effect for those with high social participation (from 0.148 to 0.114).

These findings suggest that the interaction between emotional support and social participation may play a role in reducing arthritis risk, particularly among individuals with higher levels of social participation.

## 4 Discussion

Using data from a nationally representative epidemiological survey of 15,854 older adults, this study examines the association between social factors and the prevalence of arthritis. First, we found significant differences in demographic, physiological, and psychological variables between older adults with and without arthritis. In terms of demographic variables, women and individuals with lower incomes had a higher prevalence of arthritis, consistent with previous findings (16–18). Age and low education levels were negatively correlated with self-reported arthritis prevalence. One possible explanation is that advanced age reduces patients’ willingness to seek medical attention. Additionally, previous studies have suggested that lower education levels may hinder the acquisition of health knowledge, thereby decreasing disease recognition rates (19).

Regarding physiological indicators, older adults in the arthritis group had a higher BMI and poorer daily functioning compared to those without arthritis, aligning with prior research (20–22). From a psychological perspective, consistent with previous studies, the arthritis group exhibited higher levels of anxiety and depression (23). Interestingly, they also had better cognitive performance, which may be because older adults with better cognitive function are more likely to accurately self-report their arthritis status.

The main findings of this study indicate that three social factors are significantly associated with the incidence of arthritis. Specifically, a greater number of cohabitants is associated with a lower self-reported prevalence of arthritis, suggesting that a larger social network may serve as a protective factor against arthritis. This is consistent with previous findings that, among older adults, a larger social network contributes to better physical and mental health, particularly in relation to inflammatory responses (8, 24, 25). Additionally, in multi-person living environments, older adults are more likely to receive assistance in seeking medical care and undergoing routine check-ups (26), which also contributes to the prevention of arthritis.

Moreover, this study found a significant positive correlation between high levels of social participation and the incidence of arthritis. The assessed social participation included collective physical activities (e.g., group exercises) and social behaviors (e.g., chatting, card games). Regarding the impact of physical activity on joints, a review study found inconsistent conclusions in previous research. A previous review (27) highlighted the inconsistent conclusions in the literature regarding the impact of physical activity on joints. Some studies suggested that participation in group sports was a risk factor for osteoarthritis, while others found that increased physical activity was a protective factor for hip and knee arthritis. The group activities included in this study, such as square dancing and Tai Chi, generally involved prolonged durations. Research indicated that excessive physical activity might increase the risk of joint health (28). Therefore, in future studies, the duration of participation in group activities could be considered as a design factor. This may explain why we observed a positive association between social participation and arthritis, as prolonged group activities could contribute to joint overload in older populations. Furthermore, behaviors such as chatting and playing cards could lead to prolonged sitting, and studies showed that sedentary behavior was a risk factor for arthritis (29). However, in our sample, no significant differences were observed between sedentary and non-sedentary social activities in relation to arthritis risk. This discrepancy may be explained by differences in measurement granularity, context, or the duration of specific activities. Future research could further investigate these mechanisms using more detailed and objective activity data. As a cross-sectional study, our design only established associations. An alternative explanation was that older adults with arthritis often experienced heightened loneliness, potentially increasing their motivation for social participation—a reverse-causality scenario requiring longitudinal verification.

Typically, the measurement of social support was divided into two dimensions: emotional support and instrumental support (30). This distinction also applied to the current study. A large body of research found that these two types of support had different effects on the physical and mental health of older adults. However, more studies seemed to indicate that, compared to instrumental support, emotional support had a more significant impact on the health of older adults. For instance, among hospitalized older adults, emotional support had a significant negative correlation with depressive symptoms (31), while instrumental support alone could not enhance the well-being of both the provider and the recipient unless emotional support was also offered (32). In bereaved older adults, high levels of emotional support had a stronger protective effect on mortality risk than instrumental support (33). This study found that emotional support was significantly negatively correlated with the incidence of arthritis in older adults, further verifying the importance of emotional support in the physical and mental health of the older adult. The results of the interaction analysis in this study also showed that, even for individuals with high levels of social participation, the risk of arthritis could still be reduced under conditions of high emotional support. One possible explanation is that emotional support can mitigate the psychological and physiological stress associated with social activities, such as by reducing anxiety and lowering inflammation levels (34). This suggested that when providing social support to older adults, greater attention should have been paid to emotional support, rather than focusing solely on instrumental support.

The findings of this study have important implications for the prevention and management of arthritis in older adults. First, while social participation benefits mental health, excessive engagement in high-intensity or prolonged physical activities should be avoided. In particular, older adults with arthritis should choose appropriate forms of exercise based on their individual health conditions. Second, emotional support plays a crucial role in arthritis management. Family members and caregivers should pay greater attention to the emotional needs of older adults rather than focusing solely on providing instrumental assistance.

This study has several limitations. First, since it uses a cross-sectional design, it is not possible to infer the causal relationship between social factors and arthritis. Although the study reveals an association between social factors and arthritis risk, it cannot determine whether social factors lead to the onset of arthritis or if arthritis itself impacts an individual’s social activities and support. This opens the possibility of reverse causality, where individuals with arthritis may increase their social participation as a coping mechanism to address emotional or physical challenges. Future research should incorporate longitudinal data to verify causal relationships and clarify the temporal order of these associations. Second, arthritis diagnoses were based on self-reports from the participants, which could lead to information bias if participants either underestimated or overestimated their arthritis condition. Additionally, due to the use of secondary data from the open-access CLHLS, the survey lacked clinical validation or detailed differentiation between arthritis subtypes such as osteoarthritis and inflammatory arthritis. This limitation may affect the precision of our findings, as these subtypes may differ in their underlying mechanisms and social determinants. Future studies incorporating clinical diagnoses or biomarkers are recommended to explore subtype-specific associations more thoroughly. Third, while the CLHLS includes several dietary-related items (e.g., staple food types, frequency of fruit and vegetable intake, cooking oil preferences), these variables are relatively broad and heterogeneous. We therefore did not include them in our main models due to concerns about construct validity and model stability. As a result, residual confounding by unmeasured factors (such as diet, genetic predisposition, or other lifestyle behaviors) may still exist. Future research using more standardized and comprehensive measures is needed to better account for such potential confounders. Finally, the social support data in this study were primarily collected through questionnaires, which may be subject to social desirability bias. For instance, older adults might be inclined to report higher levels of social support to meet societal expectations. Future research could combine objective indicators, such as actual social interaction records, to improve the accuracy of the data.

## 5 Conclusion

This study examines the relationship between social factors and the risk of arthritis in older adults. The results indicate that a larger social network is associated with a reduced risk of arthritis, while higher levels of social participation are linked to an increased risk. Emotional support, however, appears to have a protective effect. Notably, the interaction between social participation and emotional support was found to be a significant factor influencing arthritis risk. These findings suggest that strengthening the social network and prioritizing emotional support for older adults may be important strategies for reducing arthritis risk and improving overall health. Future research should further explore these relationships and their potential for intervention.

## Data Availability

Publicly available datasets were analyzed in this study. This data can be found at: https://opendata.pku.edu.cn/dataverse/CHADS.
