# Supplementary material for: How social factors relate to arthritis risk in Chinese older adults: population-based evidence
Source: Front Public Health. 2025 Jun 18;13:1604582. doi: 10.3389/fpubh.2025.1604582 (PMC12223137; doi:10.3389/fpubh.2025.1604582)
Supplement: Supplementary file 1 [file Table_1.DOCX]

Supplementary Table 1 Binary Logistic Regression Analysis of Factors Associated with Arthritis in Older Adults (Including covariates)

| **Variable** | **B** | **SE** | **Wald** | ***P*-value** | **OR (Exp(B))** | **95% CI for OR** |
| --- | --- | --- | --- | --- | --- | --- |
| Age | -0.008 | 0.003 | 9.383 | 0.002 | 0.991 | 0.985 - 0.997 |
| Gender (Male = 1) | -0.517 | 0.060 | 74.176 | <0.001 | 0.596 | 0.530 - 0.671 |
| Years of Education | 0.050 | 0.007 | 53.996 | <0.001 | 1.052 | 1.038 - 1.066 |
| Annual Household Income (1 for＞30000 Yuan) | 0.135 | 0.058 | 5.379 | 0.020 | 1.145 | 1.021 - 1.283 |
| BMI | 0.046 | 0.007 | 37.965 | <0.001 | 1.047 | 1.032 - 1.062 |
| Waist-to-Hip Ratio | 0.381 | 0.356 | 1.146 | 0.284 | 1.463 | 0.729 – 2.937 |
| Disability in ADL (Yes = 1) | 0.228 | 0.064 | 12.863 | <0.001 | 1.256 | 1.109 - 1.423 |
| Subjective Health | -0.329 | 0.035 | 89.838 | <0.001 | 0.719 | 0.672 - 0.770 |
| Anxiety Score | 0.024 | 0.010 | 5.648 | 0.017 | 1.024 | 1.004 - 1.045 |
| Depression Score | 0.009 | 0.006 | 0.406 | 0.121 | 1.009 | 0.998 - 1.021 |
| Cognitive Function (CSI-D) | 0.034 | 0.012 | 8.613 | 0.003 | 1.035 | 1.011 - 1.058 |
| Number of Cohabitants | -0.038 | 0.016 | 5.879 | 0.015 | 0.963 | 0.934 - 0.993 |
| Social Participation | 0.026 | 0.010 | 7.210 | 0.007 | 1.027 | 1.007 - 1.047 |
| Emotional Support | -0.038 | 0.014 | 7.642 | 0.006 | 0.963 | 0.937 - 0.989 |
| Instrumental Support | 0.014 | 0.021 | 0.486 | 0.486 | 1.014 | 0.974 - 1.056 |
| Constant | -2.028 | 0.515 | 15.523 | <0.001 | 0.132 | - |
